# Reply: Soluble oligomers or insoluble fibrils?

**DOI:** 10.1007/s00401-023-02634-5

**Published:** 2023-09-21

**Authors:** Anastasie Mate de Gerando, Noe Quittot, Matthew P. Frosch, Bradley T. Hyman

**Affiliations:** 1 Department of Neurology, Massachusetts General Hospital, Boston, MA USA; 2 Harvard Medical School, Cambridge, MA USA

The identification of fibrillar and non-fibrillar bioactive forms of tau is an important issue in our understanding of the mechanism of diseases such as Alzheimer disease. The Stern and Selkoe commentary expresses concerns about the idea that non-fibrillar oligomeric tau exists, or is bioactive. We here review structural (atomic force microscopy), compositional (mass spectroscopy), biochemical, and functional data as well as unpublished results from our lab characterizing oligomeric and fibrillar tau, clarify a variety of technical issues, and cite an extensive and robust literature from tau labs across the world to support the thesis that oligomeric tau, as well as fibrillar tau, could contribute to tau pathobiology.

Because of the widely different extraction conditions, aqueous phosphate-buffered saline (PBS) homogenates and sarkosyl detergent insoluble fractions clearly enrich for different forms of tau. Beyond the long-standing use of these preparations in the tau field, and the marked differences between extraction conditions, we present substantial data in this [23] and other manuscripts [3, 5, 14, 28, 31–33] which demonstrate that tau present in the PBS fraction (referred to as HMW tau in our manuscript) is different than the fibrillar, classical paired helical filament (PHF) tau in the sarkosyl insoluble fraction, yet they are both bioactive (and, as shown in the current work, similarly bioactive). Multiple lines of evidence support this:

- 1) In our original 2015 description of the bioactive HMW fraction, we showed that it was detected by the oligomer-specific antibody T22, disappeared after urea incubation, could be detected in the interstitial fluid from mouse brain, and we demonstrated (by atomic force microscopy) that tau immunoprecipitated from the HMW fraction contained small globular structures, all consistent with the behavior of oligomeric species of tau [32]. Atomic force microscopy was also utilized by Kayed’s group to conclusively demonstrate a brain-derived oligomeric non-fibrillar tau species [20], isolated by size exclusion chromatography and immunoprecipitated by oligomer-specific T22. This non-fibrillar preparation also demonstrated propagation and seeding [20]. The atomic force microscopy studies show, conclusively, that bioactive tau in these preparations is structurally distinct from PHF tau.
- 2) By mass spectroscopy, the aqueous extract and the sarkosyl insoluble fraction are definitively different species with similar but distinct post-translational modifications ([5, 33] and Kumar et al. submitted). The tau present in the HMW fraction enters a blot and shows a smear of high-molecular-weight material on nondenaturing Western blots, which collapse to standard bands on a denaturing Western blot—unlike expectations for fibrillar tau. Additionally, in our manuscript, we show that HMW tau is significantly less resistant to proteinase K than SARK tau, which, as noted below, is consistent with the data from the Iqbal lab [21]. Thus bioactive tau in the aqueous extract is biochemically distinct from PHF tau.
- 3) Stern may have misinterpreted our brief methods section, and we are happy to expand on the details here. To clarify, we used two types of size exclusion columns (rather than the one mentioned)—a 25 mL column for small preps (referenced in the manuscript) and a 120 mL column [HiLoad 16/600 Superdex 200pg column (no. 28-9893-35, GE Healthcare)] for larger preps (matching the sample preparation in the manuscript). For example, for the 120 mL column, the void volume is 36 mL (approximately 30% of the column volume) and we used a nomenclature in which we start collecting fraction 1 at an elution volume of 37.5 mL. We collect 2.5 mL fractions and high-molecular-weight (HMW) tau appears in fractions 2, 3, and 4, near but not including the void volume. The amount of tau in individual fractions 2, 3, and 4, as well as seeding, does not drop off in each subsequent fraction—unlike expectations if the seeding in these fractions was residua from the void volume.

We reason that if the bioactivity of the HMW fractions was simply contamination by a small amount of fibrillar species, one would expect the two preparations to act qualitatively the same. However, since the PBS sample is at worst a non-purified version of fibrils, the sarkosyl insoluble concentrated fibrillar preparation would be expected to be more robust. Yet, they are equal, or, in some parameters, the PBS fraction is even more potent, and has additional characteristics compared to the sarkosyl insoluble preparation, both in our hands and in many other labs.

- 4) Electron microscopy (EM) was performed on the sarkosyl insoluble SARK tau material, and the PBS soluble HMW tau extract (after SEC and concentration of the high-molecular-weight fractions 2, 3, and 4 by ultracentrifugation) and not the 10,000 g PBS supernatant as stated by Stern and Selkoe. Equal amounts of total tau—quantified by WB—were loaded onto the EM grids (3 µl at 0.1 µg/µl tau).
- 5) Stern and Selkoe note that we demonstrate that the PBS soluble and sarkosyl insoluble preparations are not 100% “clean” and that some fibrillar tau is present even in the PBS fraction (Fig 1 in [23]). Not unexpectedly, some short fibrils can be observed in the minimally processed PBS fraction, in agreement with the supplemental data shown in Stern et al 2023 after a soak and spin protocol, using cryoEM [30]. Of note, we do not claim that we can observe oligomeric tau by negative stain transmission EM, which would require additional purification and optimization. Importantly, however, the absence of oligomeric forms in a cryoEM preparation, such as that reported by Stern et al. [30], is uninformative, since oligomers would likely be far less detectable, or even invisible by cryoEM, compared to the repeated structures of fibrils. Critically, we show in the manuscript (Fig S1d-f and surrounding text) that the fibrillar structures observed in the PBS fraction resolve upon sonication, but seeding activity remains unchanged, suggesting again that the main driver of the biological activity in the aqueous fraction is not the fibrillar species.
- 6) We note that published work from numerous other tau laboratories supports the idea that oligomers are distinct bioactive species rather than the “ill-defined, soluble, non-fibrillar tau species” asserted by Stern and Selkoe. Importantly, Li et al. isolated soluble, oligomeric, and sarkosyl insoluble fragments of tau from human brain by sedimentation and detergent solubility, and showed, in a manner very analogous to our study, that the oligomeric tau and the sarkosyl insoluble fractions were differentially resistant to proteinase K [21]. Both seeded tau aggregation in cell culture and induced significant tau aggregation both at the site of injection and distally in aged FVB mice [21], closely analogous to our results. The Wolozin lab similarly isolated oligomeric tau and fibrillar tau by differential ultracentrifugation, and found that both preparations led to seeding, and the characteristics of the two preparations differed [17]. Another paper from the Wolozin laboratory used PS19 mice as the source of tau, and compared oligomeric (Tris-buffered saline soluble material) to sarkosyl insoluble material (fibrillar tau) and came to similar conclusions about toxicity of oligomers, showing that the oligomeric preparation propagated across neural systems to a greater extent than fibrils [16]. These results, taken together, confirm our analyses, and support our fundamental results regarding seeding, proteinase K, and propagation of both oligomeric tau and sarkosyl insoluble preparations.
- 7) A further brief review of the recent literature shows unequivocally that soluble oligomers, in addition to, and distinct from, fibrillar forms of tau, are bioactive. These results derive from multiple labs, using multiple techniques, isolation procedures, and readouts. A recent paper from Spire-Jones shows that Alzheimer synaptic preparations contain oligomeric tau but not fibrillar tau (as assessed biochemically or by EM) and show tau bioactivity [2], extending work from the Diamond group [13] and our group that are consistent with the conclusion that Alzheimer brain contains oligomeric non-fibrillar tau [3, 31, 34] in sites such as synapses, where fibrils do not occur. Recent work from Lasagna Reeves shows that HMW tau propagates across synapses in several systems [22]. The Kayed lab has published numerous papers on the isolation, biochemical characteristics, and toxic properties of soluble oligomeric tau including recent work, showing that it drives cellular senescence [8–10, 25]. Tau transfer from cell to cell, as demonstrated for example by the Kosik lab in their recent Nature paper, is via a soluble form of tau rather than a fibrillar form [27]. Diamond’s recent work suggests that even single molecules of misfolded tau, which are certainly not fibrillar forms, are sufficient to induce misfolding and aggregation [24]. Work done independently by Kayed [25], Frost [29], Westaway [18], Livesey [26], and our lab [6], all suggest that soluble misfolded tau binds molecules at the nuclear membrane to induce toxicity. The Mandelkow lab has demonstrated that non-fibrillar pro-aggregant tau has a clear impact on neural system function [11] which we have also seen in both a fibril forming line (tg4510) and in tg21221 mice that do not develop aggregates [1]. Very recently, super-resolution microscopy has been used to detect submicroscopic small globular aggregates of oligomeric tau that are distinct from tau fibrils [4, 12]. Thus, the established literature shows that oligomeric and fibrillar tau are distinct structural and biochemical species, and both are bioactive.
- 8) Finally, ongoing work in our laboratory provides further insight into the question of fibrillar vs non-fibrillar tau species. In unpublished studies, we have further purified the material that comes off the early fractions of the SEC column, and examined its properties on ion-exchange columns. The bioactivity and tau immunoreactivity elute in fractions expected if they interact with the column, consistent with them being smaller, oligomeric forms. Accordingly, transmission electron microscopy of the bioactive eluate does not contain any fibrils. In ongoing work using an in vivo amplification protocol, we find that the HMW tau fraction does not generate bioactive fibrils, whereas sarkosyl insoluble tau does.

We agree with Stern and Selkoe that the question of which species of tau are bioactive is vital, and that careful comparison of different preparations is a key approach to improve understanding of which characteristics of tau are important for its propensity to induce aggregation, propagate across neural systems, and cause neurotoxicity. Our study does exactly that, comparing two widely used preparations, derived from the same brain tissue, to one another, and coming to the conclusion that the classical fibrillar tau that is enriched in sarkosyl insoluble fractions, and a form of tau that is soluble in just PBS homogenate, appears by native gel and by SEC column to be “high molecular weight” and thus potentially oligomeric, can both lead to the evolution of AT8-positive cells at the injection site and distal to it, and, at least for the PBS fraction, gliosis.

These data reinforce and provide orthogonal information to our previous observations from human cases that the characteristics of this PBS soluble fraction differ across cases by mass spectroscopy and correlate with rate of disease progression [5, 28]. Since cryoEM structures of fibrils are the same across cases [7], we postulate that these correlations are best explained by differences in the structure of non-fibrillar, but still bioactive species. No doubt further work will help clarify the critical aspects of how tau differs across cases, and while the exact mechanisms of how those differences lead to correlations with rate of disease progression remain to be discovered, our work, as well as others, suggest strongly that classically defined fibrils [19], small fibrils [15], and more recently highlighted oligomeric species [5, 28] each play a role [14]. Understanding how will be both fascinating and critically important.
